# The Devil they Knew: Chemical Documents Analysis of Industry Influence on PFAS Science

**DOI:** 10.5334/aogh.4013

**Published:** 2023-06-01

**Authors:** Nadia Gaber, Lisa Bero, Tracey J. Woodruff

**Affiliations:** 1 University of California, San Francisco, US; 2 University of Colorado Anschutz Medical Campus, US

**Keywords:** PFAS, chemical policy, environmental health, commercial determinants, industry documents, research ethics

## Abstract

**Background:**

Per-and polyfluoroalkyl substances (PFAS) are a class of widely-used chemicals that persist in the environment and bioaccumulate in humans and animals, becoming an increasing cause for global concern. While PFAS have been commercially produced since the 1940s, their toxicity was not publicly established until the late 1990s. The objective of this paper is to evaluate industry documents on PFAS and compare them to the public health literature in order to understand this consequential delay.

**Methods:**

We reviewed a collection of previously secret industry documents archived at the UCSF Chemical Industry Documents Library, examining whether and how strategies of corporate manipulation of science were used by manufacturers of PFAS. Using well-established methods of document analysis, we developed deductive codes to assess industry influence on the conduct and publication of research. We also conducted a literature review using standard search strategies to establish when scientific information on the health effects of PFAS became public.

**Results:**

Our review of industry documents shows that companies knew PFAS was “highly toxic when inhaled and moderately toxic when ingested” by 1970, forty years before the public health community. Further, the industry used several strategies that have been shown common to tobacco, pharmaceutical and other industries to influence science and regulation – most notably, suppressing unfavorable research and distorting public discourse. We did not find evidence in this archive of funding favorable research or targeted dissemination of those results.

**Conclusions:**

The lack of transparency in industry-driven research on industrial chemicals has significant legal, political and public health consequences. Industry strategies to suppress scientific research findings or early warnings about the hazards of industrial chemicals can be analyzed and exposed, in order to guide prevention.

## Key Messages

### Implications for policy makers

1. This paper analyses how the chemical industry, using industry documents, delayed disclosing the harms of PFAS, costing billions of dollars in health and environmental damages globally.
2. Many countries are pursuing legal and legislative action to curb PFAS production that may be aided by the timeline of evidence presented here.
3. The production of chemical toxicity research should be in the best interest of protecting the public’s health, including designing the research question, funding studies, and publishing favorable and unfavorable findings.
4. Legal settlements against chemical manufacturers should include documents disclosure in order to ensure transparency and accountability for industries and their products.
5. Public health and environmental policy makers should move towards precautionary principles of chemical regulation.

### Implications for the public

This paper examines previously secret documents held by DuPont and 3M, the largest manufacturers of PFAS, also called “forever chemicals.” We show how the chemical industry used the tactics of the tobacco industry to delay public awareness of the toxicity of PFAS and, in turn, delayed regulations governing their use. PFAS are now ubiquitous in the population and environment. Consumer awareness can advance calls for safer products by demanding publicly available studies of harm. Public pressure can also influence legislators to pass more health-protective environmental and chemical regulations.

## Background

Per- and polyfluoroalkyl chemicals (PFAS) have unique chemical properties that impart oil and water repellency, temperature resistance, and friction reduction. As such, they have been used in a wide range of commercial applications since the 1940s—including non-stick cookware, fabric treatment, and food packaging—as well as military and industrial uses, particularly in insulation and fire suppressants [1]. PFAS chemical properties also make them highly resistant to degradation, both in the environment and in human bodies, and they have been found in multiple environmental media globally and measured in populations in the U.S., Europe, and China [2,3,4,5,6]. Exposures continue after phaseouts; in the U.S. over 90% of pregnant women are still exposed to perfluorooctanoic acid (PFOA) and perfluorooctane sulfonic acid (PFOS) [7] despite PFOS being phased out of production and use in 2002, and, by the end of 2015, U.S. manufacturers had eliminated PFOA emissions and product content [8].

Two of the 12,034 known PFAS variants, PFOS and PFOA [9] (formerly known as C8), have been shown to adversely impact a range of health outcomes [1,10,11,12]. Two major companies in the U.S. have manufactured the majority of legacy and emerging PFAS: 3M, makers of Scotchguard, and E.I. du Pont de Nemours & Company, known as DuPont, makers of Teflon. PFAS were long presumed to be biologically inert [13], but legal disclosures and investigative reporting have uncovered evidence that companies that manufacture PFAS learned of their toxic effects on human health and the environment long before the public health community. Chemours’ website still claims PTFE is “inert to virtually all chemicals and considered the most slippery material in existence [14].” An initial lawsuit filed in 1998 (Tennant v. E.I. du Pont de Nemours & Company) ended in settlement in 2001, establishing that DuPont dumped more than 7,100 tons of PFOA-laced sludge onto Plaintiff Tenant’s property and the Ohio River, where the chemical seeped into the ground and entered the Ohio River and local water sources [15]. Legal discovery in *Tennant* showed that DuPont and 3M had evidence of clear health and environmental impacts as early as 1976 [15]. Based on this evidence, in 2004, the Environmental Protection Agency (EPA) pursued DuPont for violations of the Toxic Substances Control Act (TSCA) Section 8(e), which requires companies to report substantial risks about chemicals they manufacture, process, or commercially distribute, for failure to disclose their findings regarding PFOA, claiming the company “knew or should have known” that these compounds were toxic. The resulting settlement of $10.25 million plus $6.2 million for supplemental environmental projects was the largest civil penalty ever obtained in the U.S. under environmental statues at that time [16]. This is less than 2% of the $1 billion per year in revenues from PFOA or C8 that DuPont disclosed in its Securities and Exchange Commission (SEC) filing in 2005 [17]. A nationwide toxic tort case was filed in 2018 by ten U.S. states against manufacturers, including 3M and DuPont, on behalf of U.S. residents who were exposed to PFAS. If the class is certified, it will become the largest class action case in U.S. history [18,19].

Human harm, including pregnancy-induced hypertension, kidney and testicular cancers, and ulcerative colitis, from PFOA was not publicly established until 2011, following the reports of the C8 Science Panel, mandated and funded as part of a settlement agreement in the case of Jack W. Leach, et al. v. E.I. du Pont de Nemours & Company (no. 01-C-608 W.Va., Wood County Circuit Court, filed 10 April 2002) [15,19,20,21]. Personal injury claims resulting from the findings have totaled $670.7 million for more than 3,500 people to date [22].

In addition to the findings of the C8 Science Panel, systematic reviews show that there is sufficient evidence that developmental exposure to PFOA restricts fetal growth [23], associated with dyslipidemia (a risk factor for adverse cardiovascular effects) [12], and poses “an immune hazard” to humans, including impaired vaccine response [24]. There is also growing evidence suggestive of impacts on human reproduction and development—including infertility, premature birth, trouble breastfeeding, delayed puberty, earlier menopause, and diverse metabolic impacts [25,26]—as well as suggestions of a relationship with neurologic and behavioral disorders (including attention deficit hyperactivity [ADHD], autism, and schizophrenia) [27,28].

There are no consistent approaches to regulating exposures to PFAS globally. For example, no federal enforceable limits on any PFAS chemicals have been established in the U.S. In 2016 EPA issued a non-enforceable lifetime Health Advisory for exposure to PFOA and PFOS in drinking water of 70 parts per trillion [26]. As of November 2015, the approximate safe level of PFOA in water systems was exceeded by factors of 100 or more in 27 U.S. states [29,30]. EPA announced a proposed National Primary Drinking Water Regulation (NPDWR) for six PFAS including perfluorooctanoic acid (PFOA), and perfluorooctane sulfonic acid (PFOS) in 2023 [31]. Monitoring of PFAS in drinking water systems remains elusive, however, because a national program to test for them ended in 2015, and even then, tested for only a few types of the thousands of chemicals, requiring only systems that serve more than 10,000 people to report only if levels exceed the threshold level. Subsequent testing has found PFAS is widely present in U.S. drinking water, as well as groundwater and rainwater [32,33]. Independent scientists argue that even this figure is too high to be health-protective and have suggested a limit of 1 ppt [34]. In light of this evidence, and ongoing research, there has been an increase in regulatory oversight and legal intervention into the use and disposal of PFAS in the United States. In December 2019, under the National Defense Authorization Act, 172 per- and polyfluoroalkyl substances were added to the federal Toxics Release Inventory (TRI), requiring monitoring of chemicals that are known to cause serious human health and/or environmental harm [35]. On June 15, 2022, EPA issued interim updated drinking water health advisories for PFOA and PFOS that reduced those EPA issued in 2016 to 0.004 and 0.020 ppt, respectively [36]. Some states in the U.S. and a few select countries globally have banned PFAS in food contact materials, paper and paper food packaging, and firefighting foam [4]. There are current efforts toward developing improved methods for measuring total PFAS in certain media [4].

Previous studies of the tobacco, food, pharmaceutical and chemical industries show that industry’s influence on the design, conduct, and dissemination of research plays a strong role in shaping public health knowledge about harmful products and determining product safety standards, particularly in the U.S. market [13,37,38,39,40,41,42]. These industries have been shown to share strategies for manipulating scientific information in order to maximize their profits. Much of the evidence about corporate manipulation of comes from analysis of previously secret industry documents that have been released through litigation [37]. This influence, and its distorting effects on public health science, is increasingly considered a major etiology of disease, particularly in advanced industrial societies [43,44,45]. A growing body of literature describes it as part of the commercial determinants of health [43,44,45].

In this paper, we analyze previously secret industry documents to: 1) examine what chemical manufacturers knew about PFAS and their effects on human health and 2) establish when they knew it in comparison to public knowledge of their health effects. We also analyze the strategies used to manipulate the science narrative. We hypothesize that, similar to the tobacco and other industries, the two largest manufacturers of PFAS, DuPont (makers of Teflon) and 3M (makers of Scotchguard), were aware of the hazards of PFAS long before the public health community and used some of the same strategies that other industries have used to sustain doubt about the dangers of their products [42]. Our review of this small collection provides a solid framework on which scholars can build as more documents and data become available. To our knowledge, this is the first use of chemical industry documents in a scholarly evaluation of the influence of the chemical industry on PFAS science and regulation.

## Methods

From September 2020 to October 2020, we organized and analyzed industry documents regarding chemicals of the PFAS family, archived in special collections of the UCSF Chemical Industry Documents Library (CIDL). The CIDL is part of a larger library of documents from tobacco, food, drug, and fossil fuel industries that influence public health, growing from donation of key documents from the tobacco Master Settlement Agreement (1998) into one of the world’s largest archives of its kind [46]. Our first aim was to compare reports and comments on health research from the internal documents to the public record, establishing a comparative timeline. Our second aim was to evaluate the strategies involved in delaying or obscuring the public health knowledge of the harms of PFAS.

### Framework and Analytic Approach

Much has been written about the methods used to search industry documents [47,48]. Less has been written about the methods of analysis used to interpret the documents. Researchers have drawn on interpretive methods widely used among historians and social scientists. These include qualitative techniques, where recurring themes are identified through deductive [49] or inductive reasoning [50] and grouped for discussion. For this paper, deductive codes were adapted from published studies in the literature detailing industry strategies of manipulating science, particularly White and Bero [37], which looked at strategies across five industries. Because the body of documents available is relatively small, it was possible to read each document and assess whether it matched our codes of interest.

### Data

This collection was gathered through litigation discovery by Robert Bilott, the attorney who first successfully sued DuPont over PFAS. Documents were donated to the UCSF Chemical Industry Documents Library and to makers of the film, *The Devil We Know* [51]. We reviewed all 39 documents in the collection, dating from 1961 to 2006.

#### 1. Timeline of Industry versus Public Knowledge

First, we conducted a literature review of published scientific information on the toxicity of PFAS. We searched PubMed in the date range 1940–2002 using the following terms and Boolean operators: “PFAS OR PFOA OR PFOS OR Teflon OR PTFE OR “C8” OR APFO AND toxicity” and this returned 595 results (prior to ~2015 PFAS were referred to as PFCs or PFAAs). We used 2002 as the end of the date range because that is the year the *DuPont vs. Leach* case was filed, alleging health harms due to PFAS, which led to the C8 Science Panel and an exponential rise in subsequent public health research. We used PubMed to screen by publication type, limiting our findings to research articles and systematic reviews. This review identified when certain health concerns were studied as PFAS were developed, enabling us to establish a historical timeline of public knowledge of PFAS health effects.

#### 2. Strategies of Industry Influence

Second, we conducted an analysis of industry strategies used to influence the science on PFAS. Drawing on the work of Bero and White [37], we deduced six codes from the cross-industry strategies of manipulation that researchers previously established to see whether the same practices emerge among the PFAS industry. The six strategies are: “manipulation of the research question to obtain predetermined results; funding and publishing research that supports industry interests; suppressing unfavorable research; distorting the public discourse about research; changing or setting scientific standards to serve corporate interests; and disseminating favorable research directly to decision makers and the public, bypassing the normal channels of scientific discourse.” We expanded “manipulation of the research question” to “industry influence on the research agenda,” a broader categorization established in the literature by Fabbri, Lai, Grundy, and Bero [52]. We then analyzed the documents, coding for each of these strategies. Multiple codes could be relevant to a single document. If relevant but outside these codes, we used an “other” designation, but it did not necessitate creating a new (inductive) category. We have included text excerpts to illustrate our categorization.

## Results

### Timeline of Industry and Public Health Knowledge of PFAS Exposure and Hazards

**Figure 1:**
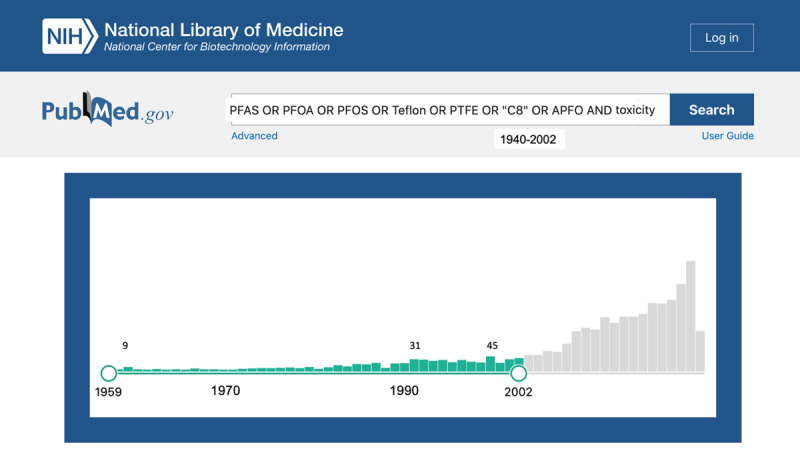
Hits from PubMed literature search of “PFAS OR PFOA OR PFOS OR Teflon OR PTFE OR “C8” OR APFO AND toxicity” from 1940–2021 (March), highlighting date range of study, 1959, the earliest hit, to 2002, the year of the Leach vs. DuPont filing.

Our PubMed search returned 595 results. Of these, only 66 were published before 1980 (Figure 1). By comparison, from 2002 to 2020, more than 2,500 papers were published.

### Public Health Knowledge of PFAS

**Figure 2:**
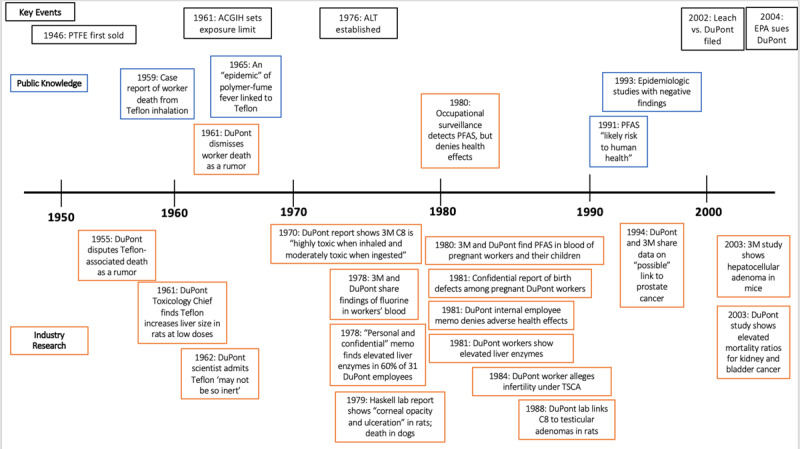
Timeline of notable public health research and health-related industry findings on PFAS health effects, with key historical events in black. The period of interest extends from 1940 to the 2002 Leach vs. DuPont filing, though we include two papers submitted by industry to the EPA after that filing with positive findings of harm and the 2004 lawsuit the EPA subsequently filed, for context. Industry research is in orange; non-industry papers are in blue. Above the timeline are papers in the public domain, and below are papers in the private domain.

**Figure 3:**
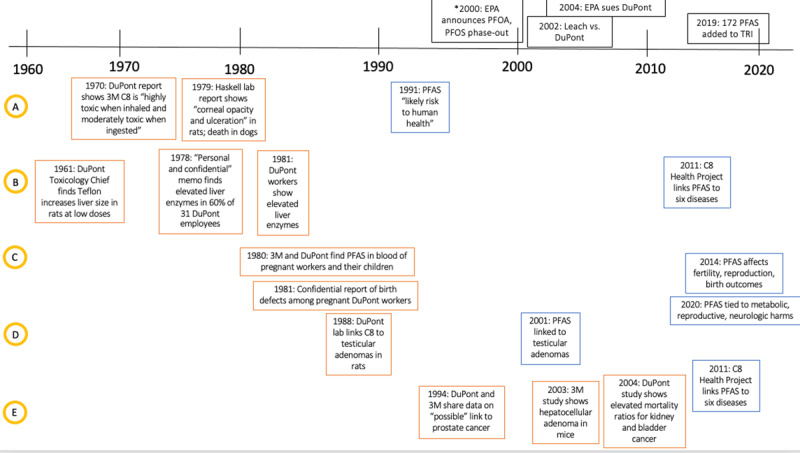
An extended timeline following five health outcomes of interest, showing when information was known by industry (in orange) and in the public domain (in blue). A = Systemic toxicity, B = Liver toxicity, C = Reproductive outcomes, D = testicular adenomas, E = Other cancer risk.

Little was publicly known about the toxicity of PFAS for the first fifty years of their use. They had been considered biologically inactive until around 2000 [53]. As shown in Figure 2, industry had multiple studies showing adverse health effects at least 21 years before they were reported in public findings. Figure 3 extends this timeline to 2020 to trace industry versus public knowledge into five key health effects of PFAS.

One of the earliest studies in our search, in 1959, examined potential respiratory and dermal toxicity from the manufacture of new plastics, including PTFE [54]. It noted that cases of “influenza-like attacks” had been reported in the occupational literature, attributed to smoking tobacco contaminated with the compound. Experimental studies of rats exposed to PTFE showed the compound was lethal to rats when heated above 300**°**C, but “no abnormality was found in other organs [55].”

In the 1950s-1970s, several case and cross-sectional reports of worker illnesses related to Teflon-contaminated cigarettes were published [55,56,57,58]. One published in the *Canadian Medical Association Journal* in 1961 [59] spurred several replies from physicians, who openly debated the dangers of Teflon decomposition under heat [60]. The case, of a worker on a U.S. base who allegedly smoked a cigarette contaminated with Teflon and died on site, was also disputed by DuPont and the Air Force as a rumor [61]. A similar release was originated from the office of the Inspector General, United States Air Force, in March 1958, and the U.S. Navy News Letter of January 1959 [61]. The author of the original paper [59] published a retraction and downplayed the respiratory hazards of Teflon inhalation as confounded by smoking writing: “The Union Carbide Corporation, upon further investigation, and with the co-operation of du Pont, reported, in December of last year, ‘there have been no deaths or permanent injuries known to stem from Teflon; *all rumors of death are false*’ (italics added) [59,62].” In a 1962 review for *Archives of Environmental Health*, Haskell Laboratories and DuPont scientist Dr. John Zapp wrote: “As a class, the plastics and resins are not as exempt from health and toxicity problems as one might have supposed them to be on the grounds of their large molecular weight and chemical inertness [61].” A 1965 paper asserted Teflon was the cause of “an epidemic of polymer fume fever [57].” Still, most believed these materials safe and uniquely useful [63], and little research was published.

In 1973, a test of the toxicity of Teflon pyrolysis products on quail and parakeets found that a fry pan coated in Teflon was lethal to the birds when heated to 280**°**C [64]. This study appeared ten years after Teflon-coated pans were introduced to the U.S. consumer market [65]. In 1980, a published occupational surveillance study by the medical director of 3M found elevated levels of fluorochemicals in blood of 3M plant workers [66]. Though the company scientist concluded there were “no ill health effects attributable to the exposure,” the persistence of fluorochemicals in workers’ blood was a new finding, which subsequent public health research investigated further.

Beginning in the 1980s, there were increasing numbers of animal toxicology studies published showing liver damage, alterations of lipid metabolism, DNA damage, and increased tumor incidence [67,68,69,70,71,72,73,74,75,76]. Based on these findings, researchers in the late 1980s proposed that the hepatic effects included cell damage that was “irreversible [68,73],” and that PFOA “may represent a severe health risk [73].” Until at least 1990, research may have been impeded because radiolabeled PFOA was not made available commercially for pharmacokinetic studies [77]. It was clear by 1980, however, that PFOA predominantly accumulates in serum, with a long half-life in humans [66]. However, a 1993 cohort mortality study found no increased mortality from liver disease [78], and a 1996 study—funded by 3M—of occupationally exposed men found no clinical hepatic toxicity, though did find abnormalities in liver enzymes that could suggest subclinical disease [79].

It was not until the early 2000s that mammalian toxicology research reignited concern with these chemicals, focused on developmental outcomes. In a 2003 study, rats exposed to PFOS throughout pregnancy birthed newborns with dose-dependent harmful effects. In one study, those in the high-dose group (10 mg/kg) were born pale and inactive, dying within an hour. At a lower dose (5 mg/kg), newborn pups became moribund but survived 8–12 hours. Over 95% of these offspring did not survive the first day of postnatal life, though no excess mortality was recorded among those that lived longer than a week [80,81]. Extended reviews of the reproductive and developmental effects of PFAS have been undertaken elsewhere [82].

### Published Industry Studies

As reported by Lau et al. [53] the toxicology of PFOS and PFOA was reviewed in a report by 3M, submitted to the EPA in 2003, which showed an increase in hepatocellular adenomas at a high dose of 20 ppm in the diet; those monitored after withdrawal of the exposure for one year showed increase thyroid follicular cell adenomas, though the reason remains unclear [83]. Short-term studies in rats and mice showed that both compounds induce peroxisome proliferation, though they did not report either to be mutagenic in a variety of assays. Increased kidney and liver weights were seen in rats given 900mg/kg for 28 days, according to an internal study [84]. A 2003 cohort study by DuPont, also filed with the EPA but not published elsewhere, shows that standardized mortality ratios were elevated and significant for bladder and kidney cancer [85]. A 2006 retrospective study also showed elevated standardized mortality ratios for bladder, kidney, and liver cancer in males, but only diabetes mellitus mortality was statistically significant [86].

## Industry strategies to Influence Science

In the UCSF Library’s PFAS collection, we identified documents supporting industry influence of science and policy in four of the six strategy areas (Table 1 and summarized below).

**Table 1:** Evidence of Industry Influence on Public Understanding of PFAS toxicity (strategies adapted from White and Bero 2010).


INDUSTRY STRATEGY	YEAR	INDUSTRY DOCUMENTS EVIDENCE

**Influence Research Question:** Industry decides what to study, or not, in order to produce evidence detracting from harms of their product.		

	1978	DuPont’s occupational physician noted “unusually high” liver enzyme elevations but dismissed findings as clinically insignificant, despite inadequate statistical power, neglecting to pursue research [87].

	1981	DuPont’s contract lab used alternate protocol to run liver enzyme samples of exposed employees; after “reevaluation” the majority of concerning tests were ruled “normal [88].”

**Fund and Publish Favorable Research:** Industry funds and publishes research that concludes their products were safe.		

	1996	3M funded a study of occupationally exposed men and found no clinical hepatic toxicity [79].

**Suppress Unfavorable Research:** Industry documents harms that are not made public.		

	1961	C6, C9, and ART increased liver size of rats even at low doses, should be handled “with extreme care [89].”

	1970	Industry Lab report finds C8 “highly toxic when inhaled and moderately toxic when injected [90].”

	1979	DuPont’s Haskell labs found “corneal opacity and ulceration” in rats, death in two dogs from ingesting APFO in low doses [91].

	1981	Record of two children born to exposed workers with eye and facial defects; PFAS found in cord blood in a third [92].

	1981	Confirmed fetal eye changes related to C8[93].

	1990	DuPont lab links C8 to testicular adenomas in rats [94].

	1994	3M knew of “possible” prostate cancer and shared with DuPont [95].

**Distort Public Discourse:** Industry works to distort public discourse, both within and outside the companies.		

	1980	3M internal communications says that C8 is “about as toxic as table salt [96].”

	1981	DuPont and 3M joint employee communications denies workers have been exposed at levels that could cause adverse health effects, denies adverse pregnancy outcomes [97].

	1991	DuPont public press release denies adverse health effects [98].

	2000	With Tenant lawsuit on the horizon, email from DuPont manager says, “the plant recognizes it must get public first… better late than never [99].”

	2006	DuPont demands the EPA certify Teflon as safe and deny any adverse health effects linked to PFOA [100].

**Change or Set Scientific Standards:** Industry sets occupational safety standards within the workplace as well as public safety standards.		

	1991	DuPont insisted no EPA notification was warranted, years after determining PFAS were a chronic hazard [101].

	2000	Public water utility informs customers that DuPont insists its own exposure guidelines are health protective [102].

**Targeted Dissemination:** Industry strategically disseminates information to key policymakers.		

		N/A


### Influence Research Question

In this collection, we identified two examples in which company researchers identified abnormalities in workers exposed to PFAS but repeated and redesigned the studies until abnormalities were no longer found.

DuPont’s Medical Surveillance division was asked in 1978 to undertake a review of the medical records of employees exposed to C8. Though no “unusual health problems were found” amongst the group, DuPont’s occupational physician, Y.L. Power, reports he is “disturbed by the frequency of borderline elevated liver function,” observed in 60% of the 31 employees tested. These were not considered by Dr. Power to be clinically significant, and he notes that the small number of records reviewed (31) would not make the findings statistically valid [87].

In 1978, Dr. Power reported an “unusually large percentage” of elevated liver enzymes among employees working in proximity to Teflon, nearly four times higher than control subjects working elsewhere [88]. Though the report included an internal control group in non-exposed workers, the tests were nonetheless “reevaluated” by sending them to both a “standard” laboratory (General Consultants, Inc.) and a contract laboratory (Upjohn Laboratory). Though trends for the enzymes were correlated between the two laboratories, the range of “normal” was defined differently. Dr. Power and DuPont chose to use the more permissive range of normal, leading DuPont to internally report that “there is no conclusive evidence of an occupationally related health problem among workers exposed to C-8 [88].”

### Fund and Publish Favorable Research

Our review of this documents collection did not find evidence of this strategy. We return to this in the Discussion.

### Suppress Unfavorable Research

Internal studies were identified, ranging from 1961 to 1994, showing that DuPont had evidence of PFAS toxicity from internal animal and occupational studies that they did not publish in the scientific literature and failed to report their findings to EPA as required under TSCA. These documents were all marked as “confidential,” and in some cases, industry executives were explicit that they “wanted this memo destroyed [95].”

In a 1961 report from Teflon’s Polychemicals Research and Development department, Dorothy Hood, Chief of Toxicology, evaluated the toxicity of Teflon dispersing agents in rats and found that liver enlargement was recognized as “the most sensitive sign of toxicity [89].” It found that Teflon materials had “the ability to increase the size of the liver of rats at low doses.” The report recommends that all of these materials be handled “with extreme care” and that “contact with the skin should be strictly avoided.”

In a 1970 internal information request, W.E. Hilton of the Fluorocarbons Division at the Washington Works plant notes that studies by Haskell Laboratory had already found C8 to be “highly toxic when inhaled and moderately toxic when ingested [90].” Haskell Laboratory was founded and funded by DuPont in order to study the health effects of its products and was frequently contracted by various DuPont departments to conduct toxicologic analyses [103].

In a 1979 private report for DuPont, Haskell labs performed a series of animal tests with the chemical APFO (the ammonium salt of PFOA), finding a range of toxic effects. PFAS-exposed rats showed liver enlargement at low doses, and some were observed to have “corneal opacity and ulceration” that remained for up to 42 days after. Haskell labs reported these chemicals to be “highly toxic when inhaled.” The chemical was also administered in a single dose of 450 mg to two dogs, who died two days after ingestion and who showed increased plasma enzyme level “indicative of cellular damage [91].”

In 1980, the Employee Relations Department of DuPont and 3M issued a Pregnancy Outcome Questionnaire for information about workers’ pregnancies. Results at DuPont showed that of eight pregnancies in the period of interest, two babies were born with birth defects: one with eye defects and a single nostril, and the other with eye and tear duct defects. A third child had detectable PFAS in cord blood. No other abnormalities were noted. Not only were these results not published, they were not communicated to employees, as discussed under the strategy of Distorting Public Discourse [92].

In a follow-up 3M study of C8 exposure in pregnant rats in 1981, R.E. Staples and Taisan Chiu confirmed that “observed fetus eye changes were due to C8.” A half-life of 7–9 days in male rats was compared to an earlier study at 3M that suggested the decay rate in females was “much higher.” The document explicitly describes the companies’ sharing of toxicity information and their mutual coordination with Haskell Laboratories to plan internal research indicative of cellular damage [93].

In a 1988 internal report, DuPont’s Haskell laboratories clearly lists the dangerous qualities of the chemical C8, reporting it has “moderate acute oral toxicity,” is “highly toxic” when inhaled, citing internal studies ranging from 1969 to 1981. It conveyed findings from a study in which rats were fed low- or high-dose diets of C8, resulting in “liver degeneration, enlarged livers, and increased liver enzymes in both groups.” The report also confirms that C8 produces ocular damage when exposed to the eye without washing. Subchronic studies of oral and dermal exposure “confirm the effect of C-8 on the liver,” it states, including the only citation from the peer-reviewed literature [67,104].

A 1994 DuPont report summarized that the companies had by then established the half-life of C8 in human blood of between 1.5–3 years, but that “no adverse health effects were found in 3M workers in a study of liver function in DuPont Washington Works.” Still, a “possible increase in prostate cancers” had been reported at a 3M facility manufacturing C8 [95].

### Distort Public Discourse

Following internal surveys about pregnant workers’ health and birth outcomes, DuPont decided to remove female employees from areas where they are exposed to the chemical. As noted above, they did not publish the health effect findings, nor did they communicate the problem to their employees. Instead, they made the policy change look like it was a precautionary measure, rather than a response to adverse events. In a 1981 communication memo to its employees, DuPont wrote that “during the period that C8 has been used at Washington Works there is no known evidence that our employees have been exposed to C8 levels that pose adverse health effects.” The sample Q&A of this April 1981 document says, “We know of no evidence of birth defects caused by C-8 at DuPont. In light of 3M results, we will investigate further [97].” This followed a 1980 communications memo, shared by 3M and DuPont, the companies proclaim that “There is a dose level where almost every chemical becomes poisonous, even water” and that C8 “has a lower toxicity, like table salt [96].”

DuPont also downplayed the significance of PFAS toxicity in public communications. In a 1991 draft of a public press release, responding to evidence of PFAS in groundwater near a DuPont manufacturing plant, the company repeats that “According to studies by DuPont and 3M Corporation, the manufacturer of the substance, C-8 has no known toxic or ill health effects in humans at concentration levels detected [98].”

In a series of emails, an in-house DuPont attorney, Bernard Reilly, discusses 3M’s announcement that it will no longer be manufacturing Scotchguard because “it is too persistent in the environment and gets into our blood [105].” Reilly acknowledges that “the plant recognizes it must get public first… better late than never [99].”

Following the *Tennet* and *Leach* trials (1998 and 2002, respectively), as media attention to PFAS contamination increased, DuPont attempted to work with the EPA to control the narrative. In an email, “Urgent: EPA Action Needed,” DuPont vice president Susan Stalneck writes: “We need to EPA to quickly (like first thing tomorrow) say the following” and lists two specific talking points: 1. That “Consumer products sold under the Teflon brand are safe” and 2. “Further, to date, there are no human health effects known to be caused by PFOA [100].”

### Change or Set Scientific Standards

DuPont had considerable influence over the information available to environmental regulators, municipal utilities, and their customers. The documents show the company had evidence of concern about the extent of PFAS exposure and human health risks it withheld from EPA by affirming its own health hazards analysis were sufficient.

In DuPont’s 1991 review of C8 they listed the contamination concentration from the chemical at areas in and around the Washington Works factory as well as plans for remediation. At the time its toxicology had been disclosed, its presence in the aquifer was reported to regulatory agencies, and its discharge from sites was being regulated. By 1991, 3M had set a Threshold Limit Value of 100 ug/m^3^ and DuPont set an Assigned Exposure Limit of 10 ug/m3, matching the American Conference of Governmental Industrial Hygienists (ACGIH) standard. Still, DuPont insisted that no EPA notification was warranted because “no health hazards exist based on the information we have [101].”

In 2000, the Lubeck Public Service District (LPSD) in West Virginia disclosed that a number of PFAS chemicals, including C8, used at the Washington Works factory had been detected in the drinking water. The LPSD stated that “DuPont reports that it has toxicological and epidemiological data to support confidence that exposure guidelines established by DuPont are protective of human health [102].”

### Targeted Dissemination

We did not find specific evidence of this strategy in the collection available. However, the preceding section illustrates that industry’s influence over regulatory standards involves dissemination of information directly to policymakers.

## Discussion

This collection of industry documents shows that DuPont and 3M shared toxicologic studies and knew C8 was “highly toxic” when inhaled and “moderately toxic” when ingested by 1970, well before the public health community had such information available. There is very little published literature between 1961 and 1991, though this review of documents shows that this is the time period in which DuPont and 3M seemed to become suspicious of the chemicals’ toxicity.

The documents provide a particularly illuminating glimpse into how the company managed its own employees’ concerns before making its toxicity concerns public. By 1981, DuPont had decided to remove women of childbearing age from any potential exposure to C8, a covert admission of harm. That same year, the company learned that some pregnant workers suffered miscarriages, while others birthed children born with the same birth defects found previously in animals. Birth defects attributable to PFAS were first reported in the medical literature more than thirty years after industry knew. By 1994, DuPont’s internal reports state the company should “evaluate replacement of C-8 with other, less toxic materials [95].”

Industry influence on science and regulatory agencies has been demonstrated to be an important force shaping public health. Bero and White have shown that these strategies are consistent across industries, including the tobacco, pharmaceutical, lead, and polyvinyl chloride industries (2010) [37]. Here, we provide evidence of those same strategies being used by DuPont and 3M regarding the PFAS class of chemicals. Our analysis of the PFAS collection at the UCSF Chemical Industry Documents Library shows that, with respect to PFAS, the influence of these industries is marked more by the production of silence than by the manufacture of doubt. The main strategy used was suppression of unfavorable research, a category under which we have included non-publication of evidence of harm.

The results offered here situate primary accounts of industry knowledge of PFAS harms in historical context, scaffolded on the backbone of decades of industry documents research, particularly regarding tobacco companies’ influence on science and regulation. The limitation of this study is the small number of documents available for analysis. However, because the methods applied here depend on a demonstration of tactics and strategies, rather than a quantitative review, we anticipate that our findings retain validity as new documents emerge [15]. Indeed, we expect that our findings will be strengthened as more documents become available and offer an important framework for future research.

Ongoing investigations and research into the inadequacies of chemical policy increasingly points to the role of industry in minimizing regulatory oversight, in the U.S. and around the world [106,107,108,109,110]. Public agencies in fact relied on DuPont to certify that exposures to the chemicals did not pose a human health risk because so little was known publicly about the chemicals, as shown in one of the documents from this collection [102]. Like Big Tobacco, the major chemical manufacturers have a vested financial interest in suppressing scientific evidence of the harms of their products, while maintaining the public perception that their products are safe. The U.S.’s failure to shift the burden of proof to the industry with respect to chemical policy means that we may always be chasing the devil they knew, rather than defending public health from the outset [109].

Further research is needed to examine the extent of these strategies. Making documents public should be an essential part of any settlements in ongoing litigation against DuPont, 3M, and other chemical manufacturers of PFAS, in order to understand and combat the strategies of industry influence. In the interest of public health, corporations should be held to the same standards of data sharing and open science that are increasingly adopted by independent scientists [111]. Cost of delay models could help estimate the devastating social and economic impacts of this influence. Scientists should be aware of industry efforts to subvert these principles of research integrity when evaluating chemical safety data [112]. This study builds on the scientific research into industry influence on public health, and invites acquisition of more documents, made publicly available in archives like the UCSF Chemical Industry Documents Library.
